# Pleiotropic roles of the matricellular protein Sparc in tendon maturation and ageing

**DOI:** 10.1038/srep32635

**Published:** 2016-09-02

**Authors:** Renate Gehwolf, Andrea Wagner, Christine Lehner, Amy D. Bradshaw, Cornelia Scharler, Justyna A. Niestrawska, Gerhard A. Holzapfel, Hans-Christian Bauer, Herbert Tempfer, Andreas Traweger

**Affiliations:** 1 Institute of Tendon and Bone Regeneration, Paracelsus Medical University - Spinal Cord Injury & Tissue Regeneration Center Salzburg, Austria; 2 Austrian Cluster for Tissue Regeneration, Vienna, Austria; 3 Gazes Cardiac Research Institute, Medical University of South Carolina, Charleston, USA; 4 Experimental and Clinical Cell Therapy Institute, Paracelsus Medical University Spinal Cord Injury & Tissue Regeneration Center Salzburg, Austria; 5 Institute of Biomechanics, Graz University of Technology, Graz, Austria

## Abstract

Acute and chronic tendinopathies remain clinically challenging and tendons are predisposed to degeneration or injury with age. Despite the high prevalence of tendon disease in the elderly, our current understanding of the mechanisms underlying the age-dependent deterioration of tendon function remains very limited. Here, we show that Secreted protein acidic and rich in cysteine (Sparc) expression significantly decreases in healthy-aged mouse Achilles tendons. Loss of Sparc results in tendon collagen fibrillogenesis defects and Sparc−/− tendons are less able to withstand force in comparison with their respective wild type counterparts. On the cellular level, Sparc-null and healthy-aged tendon-derived cells exhibited a more contracted phenotype and an altered actin cytoskeleton. Additionally, an elevated expression of the adipogenic marker genes PPARγ and Cebpα with a concomitant increase in lipid deposits in aged and Sparc−/− tendons was observed. In summary, we propose that Sparc levels in tendons are critical for proper collagen fibril maturation and its age-related decrease, together with a change in ECM properties favors lipid accretion in tendons.

Musculoskeletal diseases are the most common cause of severe long-term pain and physical disability. Of these conditions the majority involve injuries or pathological changes to tendons or ligaments. It is well established that the functional integrity of tendons decreases with advanced ageing, resulting in a marked increase in tendon and ligament injuries in older age groups^1^,^2^. However, despite the increasing burden and the debilitating nature of tendon injury and disease, effective therapies are – mainly because of the poor regenerative capacity of tendons - limited compared to other musculoskeletal tissues, such as muscle and bone. Further, our fragmentary knowledge of the cellular and molecular determinants underpinning the increased risk in developing tendinopathies hampers the development of novel and targeted treatment modalities. Although tendon ruptures can occur due to an acute overloading event or laceration, tendon injuries often are preceded by chronic tissue degeneration^3^, including collagen fiber disruption, hypercellularity, and chondrogenic and/or fatty inclusions^4^,^5^,^6^. It is believed that accumulating micro-damage within the extracellular matrix (ECM) leads to a gradual weakening of the tendon tissue as a result of an imbalance between anabolic and catabolic pathways favoring matrix degradation^7^. Generally, the homeostatic and regenerative capacities of various organs and tissues are progressively disrupted with ageing, which is in part attributed to a functional decline in tissue-resident stem cell populations^8^. Ultimately, these cell-intrinsic changes lead to impaired tissue function and a deficient response to injury. Indeed, a population of residing stem and progenitor cells has been identified in tendons^9^,^10^,^11^,^12^ which display functional age-related changes *in vitro*^13^,^14^. However, our understanding of the role of these stem and progenitor cells in tendon de- and regeneration, as well as the complex mechanisms underlying tendon ageing is far from complete.

In this work, we sought to identify the molecular determinants of age-associated changes in healthy-aged mouse Achilles tendon tissue. Our findings reveal profound changes in the expression of ECM-related proteins and a previously unknown role of Secreted protein acidic and rich in cysteine (Sparc; also known as BM-40 or osteonectin) in tendons. Sparc is a collagen-binding matricellular glycoprotein involved in collagen fibril assembly and procollagen processing^15^. If secreted, Sparc regulates cell–ECM interactions impacting upon cell signaling, adhesion, proliferation, migration, and survival^16^. Consistent with reported findings in other tissues^17^, the structure of the tendon ECM appears altered in Sparc−/− mice. Importantly, we provide evidence that the age-related decrease in Sparc leads to an increased lipid accumulation within the dense, collagenous tendon tissue, a pathology commonly seen in the elderly^18^.

## Results

### Differential screening and gene expression analysis

**Figure 1:**
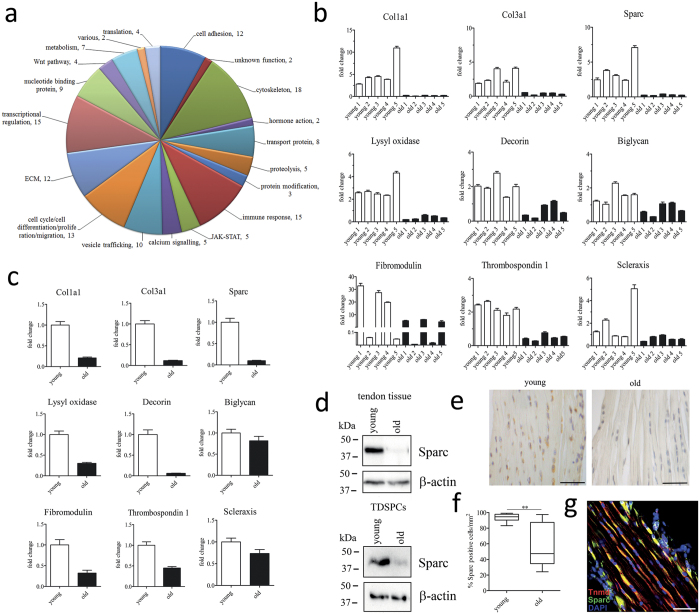
Age-related gene expression in mouse Achilles tendons. (**a**) Shown are assigned gene ontology groups for a total of 168 differentially expressed genes identified by SSH in healthy-aged C57BL/6 mouse Achilles tendons. (**b**) Genes coding for extracellular matrix proteins (e.g. Col1a1, Col3a1, Sparc, Lysyl oxidase, Decorin, Biglycan, Fibromodulin, and Thrombospondin 1) are significantly repressed in healthy-aged Achilles tendons. A moderate repression was also observed for the tendon progenitor cell marker Scleraxis. Bars represent mean ± SEM (for 5 individual animals); a fold change of ≥2.0 was assumed as a significant change. (**c**) Age-related repression of these transcripts is largely maintained in isolated tendon progenitor/stem cells of healthy-aged mouse Achilles tendons (passage = 3) *in vitro*. Bars represent mean ± SEM (n = 3). (**d**) Western Blot analysis of Sparc expression in young and healthy aged Achilles tendons (top) and TDSPCs isolated thereof (bottom). (**e**,**f**) Representative immunohistochemical images of young and healthy-aged mouse Achilles tendons stained for Sparc demonstrating a significant reduction in Sparc-postive cells in healthy-aged tendons (n ≥2500 cells per group); Scale bars: 100 μm; **P < 0.01, t-test (Mann-Whitney test). (**g**) Sparc co-distributes with tenomodulin-positive cells within the tendon proper of young Achilles tendons. Scale bar: 50 μm.

To assess age-related changes in gene expression, we performed a suppression-subtractive hybridization screen using RNA isolated from Achilles tendon tissue harvested from 3 month (young) and 18 month old (healthy-aged) male C57BL/6 mice. In total, 168 cDNA clones were isolated, and based on sequencing data, functionally annotated using DAVID^19^. Several of the identified gene ontology (GO) terms relate to genes encoding proteins important for processes such as cell adhesion, cytoskeletal re-arrangements, and cell migration (Fig. 1a). Furthermore, several genes encoding extracellular matrix proteins and polypeptides relevant for the post-translational modification of collagen were identified. Based on the sequencing data and functional clustering, the change in expression of 90 candidate-genes was then validated by quantitative RT-RCR (Table S1). Overall, the expression of the ECM and ECM-remodeling genes was significantly reduced in healthy-aged tendon tissue and tendon derived stem/progenitor cells (TDSPCs) isolated from Achilles tendon tissue (Fig. 1b/c and Fig. S1a/b). Also, mRNA levels of the tendon-related transcription factors mohawk and scleraxis, both positive regulators of collagen type I^20^,^21^, were lower in healthy aged tendons.

An age-related decrease in the expression of several of the identified genes, including collagen type I (Col1a1) and type III (Col3a1), fibromodulin (Fmod), and matrix metalloproteinases 2/9, has been previously reported^13^,^22^,^23^. However, our screen also revealed a reduction in secreted protein acidic and rich in cysteine (SPARC; BM-40; osteonectin) in healthy-aged tendon, which to our knowledge, has not been reported thus far (Fig. 1b/d top). The change in gene expression was also confirmed on the protein level from isolated Achilles tendon and TDSPC lysates (Fig. 1d bottom). We further demonstrate a significant reduction in the number of Sparc expressing cells within the dense connective tissue of healthy-aged mouse Achilles tendons (Fig. 1e/f). Finally, Sparc is co-expressed in cells positive for tenomodulin (Tnmd), a marker for tendon- and ligament-resident cells (Fig. 1g).

Taken together, next to an age-related change in expression of several ECM genes, these results demonstrate a significantly reduced expression of the collagen-binding matricellular protein Sparc in healthy-aged tendons.

### Multiscale analysis of tendons from Sparc−/− mice

**Figure 2:**
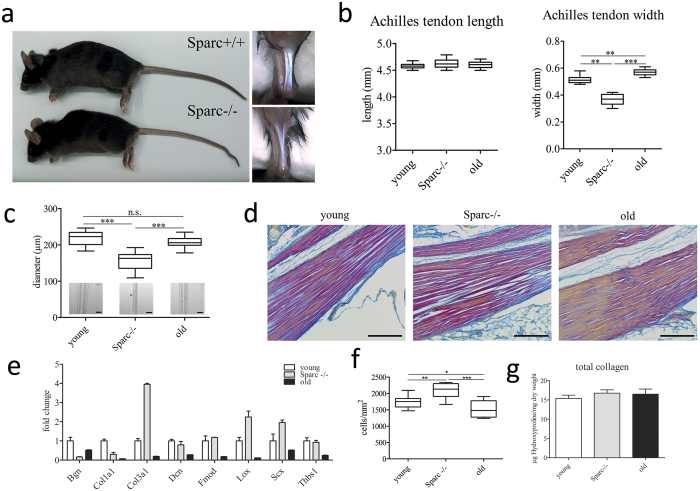
Characterization of C57BL/6 Sparc−/− tendons. (**a**,**b**) Phenotype of wild type and Sparc−/− C57BL/6 mice and Achilles tendons. Sparc−/− mice are similar in size and body weight. Achilles tendons of Sparc−/− mice are significantly thinner, but not shorter compared to their wild type counterparts (n = 10 for each group). ***P < 0.001, **P < 0.01, One-way ANOVA (Tukey’s Multiple Comparison Test). (**c**) Similarly, tail tendon fascicles of Sparc−/− mice are thinner, whereas no differences were detectable between the fascicle diameters of young and healthy-aged tail tendons. Shown are measurements of 10 tail tendon fascicles of 10 individuals each (n = 100). ***P < 0.001, **P < 0.01, *P < 0.05 non-parametric repeated Measures ANOVA (Friedman Test with Dunn’s post hoc test). (**d**) Herovici’s collagen stain of young, Sparc−/−, and healthy-aged mouse Achilles tendons showing collagen type I in red and Collagen type III in blue (scale bar: 200 μm). (**e**) Gene expression analysis of ECM-related genes in Achilles tendon tissue. Shown is fold-change in expression compared to young tendon tissue. The expression of Col3a1 mRNA is up-regulated in Sparc−/−, leading to a shift in the ColI/ColIII ratio (Figure S2). (**f**) Determination of total cell number per area in young and Sparc−/− Achilles tendons, showing a significant enhanced cell number in Sparc−/− tendons. Data information: Boxplots represent cell counts determined for Achilles tendons harvested from 10 mice. A minimum of 1000 cells per animal were counted. ***P < 0.001, **P < 0.01, *P < 0.05 One-way ANOVA (Tukey’s Multiple Comparison Test). (**g**) Total Collagen content determined by the hydroxyproline content of tail tendons extracted from the three groups. Graphs represent quantifications of 3 independent biological repeats (mean  ± SEM).

The change in expression of Sparc in healthy-aged tendons led us to investigate the role of Sparc in tendons. Macroscopically, Achilles tendons of young Sparc−/− mice (3 months) appeared significantly thinner, whereas the length of the tendon was comparable to their wild type (WT) counterparts (Fig. 2a/b). In contrast, healthy-aged Achilles tendons (18 months) were moderately thicker in comparison to young and Sparc−/− tendons (Fig. 2b). Similarly, the analysis of mouse tail tendon fascicles revealed a significant decrease in diameter for young Sparc−/− mice, whereas no significant difference was observed between young and healthy-aged mice (Fig. 2c).

We then performed histological analysis to assess tendon structure. Although no gross differences in tissue architecture were evident by Herovici polychrome staining of thin sections (and H&E and alcian blue stain; data not shown), the compactness of the tendon tissue of young Sparc−/− seemed lower, potentially due to lower inter-adherence of the collagen fiber bundles (Fig. 2d). Concomitantly, a moderate decrease in cellularity was observed for healthy-aged tendons, whereas a significant increase in tenocyte number was evident in young tendon tissue lacking Sparc (Fig. 2f). Interestingly, the number of adipocytes in white adipose tissue is also increased in Sparc−/− mice^24^. Further, an increase in blue staining is evident for Sparc−/− tendon tissue, indicating that collagen type III (Col III) is more abundant^25^,^26^. This is further substantiated by an increased expression of collagen type III mRNA in young Sparc−/− tendons as evidenced by qRT-PCR (Fig. 2e). We also examined the impact of Sparc knockout on the mRNA levels of several ECM and tendon-associated genes by qRT-PCR. With the exception of biglycan (Bgn), there was no change in expression evident for the small leucine-rich proteoglycans (SLRPs) fibromodulin (Fmod) and decorin (Dcn), whereas all of them were decreased in healthy-aged tendon tissue (Fig. 2e). In comparison, a strong shift in the ColI/ColIII ratio was observed in young Sparc−/− mice tendons, with a significant increase in Col III and decrease in Col I transcripts (Fig. 2e and Fig. S2). However, the total content of collagen as assessed by colorimetric measurement of hydroxyproline is comparable between young, healthy-aged, and young Sparc−/− tendons (Fig. 2g).

**Figure 3:**
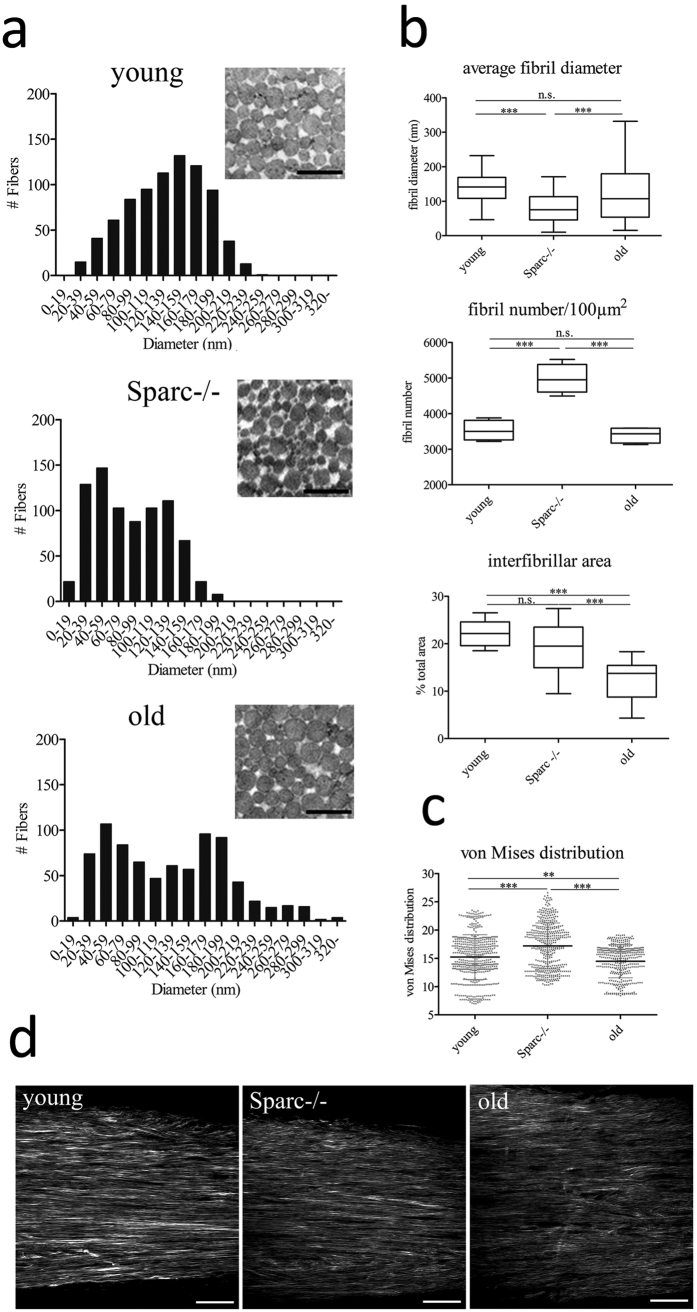
Structural analysis of healthy-aged and Sparc−/− C57BL/6 mouse Achilles tendons. (**a**) Ultrastructural analysis of mouse Achilles tendons. Shown are frequencies of fibril diameters of young, Sparc−/− and healthy-aged C57BL/6 mouse determined by TEM (n = 800 fibrils of 3 animals each). Representative images are shown in the inserts. Mean ± SEM are displayed; Scale bar: 500 nm. (**b**) The average fibril diameters are significantly different between young and Sparc−/− tendons, young and healthy-aged tendons and Sparc−/− and healthy-aged tendons. Box plots represent measurements of a total of 800 fibrils; ***P < 0.001, **P < 0.01, non-parametric repeated Measures ANOVA (Friedman Test with Dunn’s post hoc test). Fibril number per area and interfibrillar area was determined for a total of 4 animals. (**c**,**d**) Second-harmonic generation microscopy and angle analysis of Collagen fibres in young, Sparc−/− and healthy-aged Achilles tendons. Dot blots represent von Mises distributions determined from z-stacks acquired from a total of 10 Achilles tendons per group. ***P < 0.001, **P < 0.01, One-way ANOVA (Dunn’s Multiple Comparison Test); Scale bars: 50 μm.

As our data indicated a change in the fibrillar structure and/or organization, we next examined tendon tissues on an ultrastructural level using transmission electron microscopy (TEM). Overall, collagen fibrils of all groups were observed to have a uniformly circular cross-section (Fig. 3a). The average fibril diameter was moderately larger for healthy-aged tendon tissue samples and significantly smaller for Sparc−/− samples (Fig. 3b top). Also, the average diameter was more heterogeneous for healthy-aged tendons, which becomes more evident when analyzing histograms of the fibril size distribution (Fig. 3a). Qualitatively, fibril diameters appear normally distributed in young tendons, whereas Sparc−/− samples show an increase in smaller fibrils, which is in agreement with previous reports^17^. Also, a significant increase in fibril number was evident in the absence of Sparc (Fig. 3b middle). Healthy-aged tendons displayed a bimodal distribution, with a more pronounced separation of large and small diameter fibrils and less interfibrillar area (Fig. 3b bottom), showing no change in fibril number when compared to young tendons.

We next analyzed the collagen fiber orientation by second-harmonic generation (SHG) microscopy combined with an automated analysis of three-dimensional image stacks^27^. Although no dramatic differences in fiber orientation were observed, fiber orientation was lowest in healthy-aged tendon tissue, whereas the alignment was more pronounced in Sparc−/− tendons (Fig. 3c,d).

### Lack of Sparc alters tendon mechanical properties

**Figure 4:**
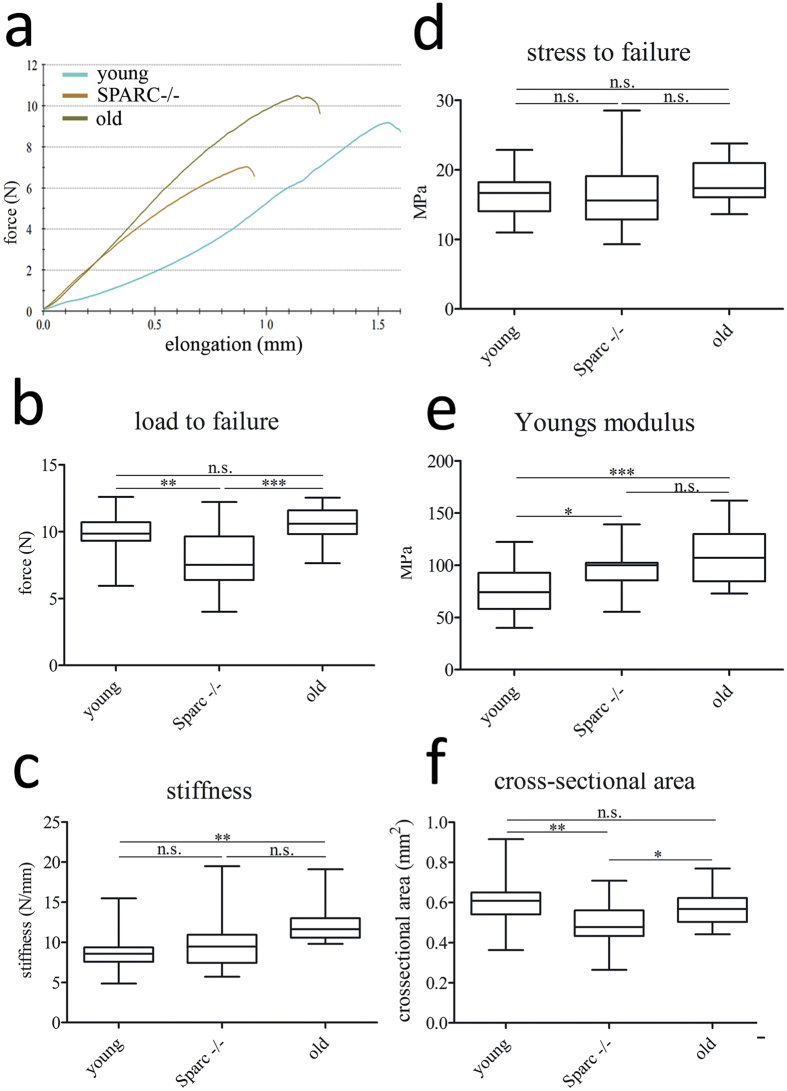
Mechanical properties of flexor tendons. (**a**) Representative force-elongation curves for C57BL/6 mouse flexor tendons of young and healthy-aged wild type and Sparc−/− animals. (**b**–**e**) Compared to young tendon tissue (n = 23), the maximum load to failure was significantly lower for Sparc−/− flexor tendons (n = 16), but not for healthy-aged tendons (n = 17). A significant difference in stiffness was only observed for healthy-aged tendons (increase), whereas the elastic modulus was increased for both, healthy-aged and young Sparc−/− tendons. However, the ultimate tensile stress (stress to failure) was comparable for all groups. ***P < 0.001, **P < 0.01, *P < 0.05 One-way ANOVA (Tukey’s Multiple Comparison Test). (**f**) Cross-sectional area of young (n = 23) and healthy-aged wild type (n = 17) flexor tendons is significantly greater compared to Sparc−/− (n = 16) flexor tendons. **P < 0.01, *P < 0.05 One-way ANOVA (Tukey’s Multiple Comparison Test).

As ultrastructural differences were evident for Sparc−/− tendons, we next evaluated the biomechanical properties of mouse flexor tendons using a tensile test. Figure 4a illustrates typical force-elongation curves for mouse flexor tendons retrieved from young, healthy-aged, and Sparc−/− mice. The maximum load to failure, indicated by the rupture point (Fig. 4a), was significantly lower for Sparc−/− flexor tendons, but not for healthy-aged tendons (Fig. 4b). However, the ultimate tensile stress at which the tendons failed was not significantly different for all three groups tested (Fig. 4d). Therefore, the mechanical properties of the tested flexor tendons were comparable and the ability of specimens retrieved from wild type animals to withstand higher forces was mainly due to their larger cross-sectional area (Fig. 4f). Finally, the change in stress per unit strain over the linear region of the stress-strain curve (not shown) – elastic modulus or Young’s modulus – was determined as a measure for the elastic deformability of the tendons. Compared to young flexor tendons, the elastic modulus was increased for Sparc−/− mice, which was even more pronounced for flexor tendons from 18 month healthy-aged mice (Fig. 4e). Taken together, although the mechanical strength of the tendons was comparable, flexor tendons from Sparc−/− and healthy aged mice were significantly stiffer (Fig. 4c/e) compared to tendons from young animals. Ultimately, due to their decreased cross-sectional area tendons lacking the expression of Sparc−/− during development and maturation are less able to withstand force.

### Aged tendon-derived cells display changes in morphology and ECM contraction

**Figure 5:**
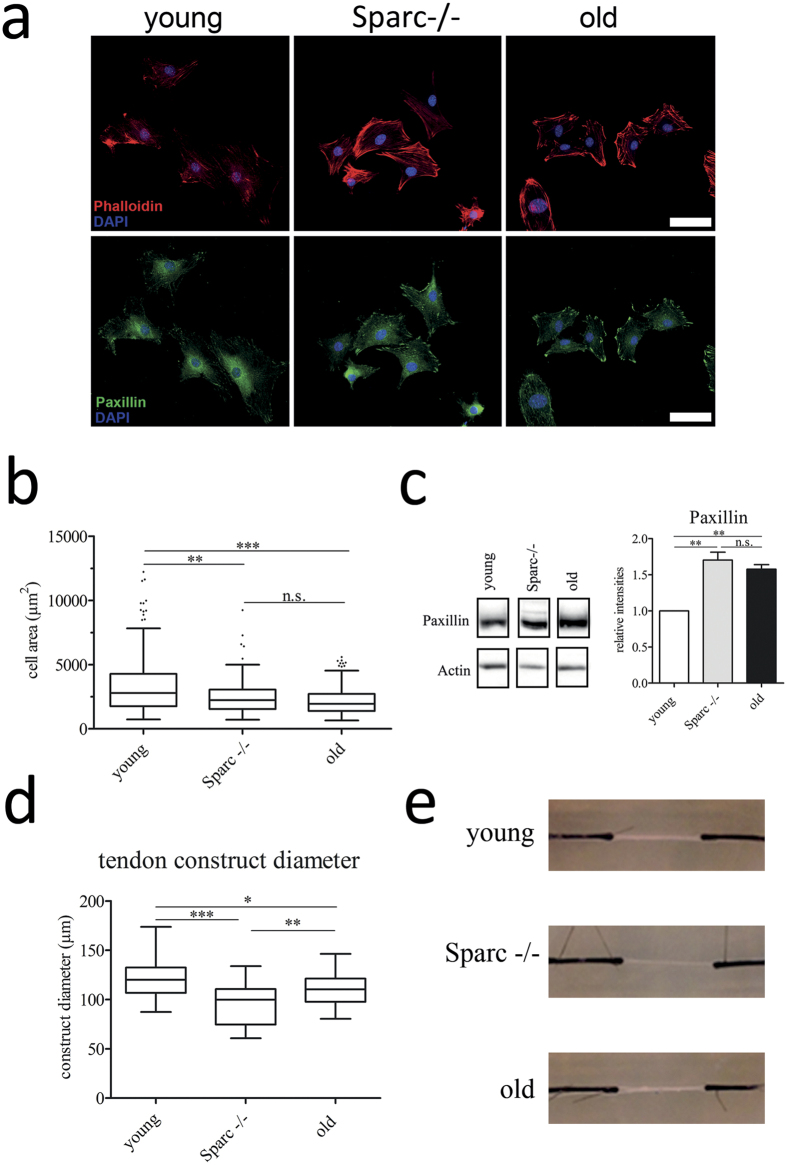
Altered tendon-derived stem and progenitor cell morphology. (**a**,**b**) Immunofluorescent staining of Achilles tendon derived cells stained for F-actin (stained with phalloidin) and for paxillin to visualize focal adhesion complexes. Scale bars: 50 μm. Boxplots represent cell surface areas determined from phalloidin-stained cells (n ≥ 340 cells; 3 independent experiments); ***P < 0.001, **P < 0.01, One-way ANOVA (Kruskall-Wallis test with Dunn’s Multiple Comparison test). (**c**) Tendon cell lysates derived from young, healthy-aged and Sparc−/− tendons were probed for paxillin, demonstrating an increase in expression for Sparc−/− and healthy-aged tendons cells. Graphs show densitometric paxillin band quantifications normalized to ß-actin expression for 3 independent experiments; mean  ± SEM; **P < 0.01, One-way ANOVA (Tukey’s Multiple Comparison Test). (**d**,**e**) Sparc−/− and healthy-aged tendon-derived cells contract *in vitro* tendon-like constructs more strongly in comparison to cells isolated from young tendon tissue. Representative images (**e**) of *in vitro* tendon constructs 42 days post-seeding are shown and graphs represent diameter measurements from 5 independent experiments; ***P < 0.001, **P < 0.01, *P < 0.05 One-way ANOVA (Tukey’s Multiple Comparison Test).

Sparc has been implicated in the regulation of cell attachment and spreading and we therefore next examined cytoskeletal elements of primary cells derived from healthy-aged and age-matched wild type and Sparc−/− mice by immunofluorescence microscopy. Figure 5a shows representative images of cells 24 hours post-seeding on collagen I-coated cover slips stained for cytoskeletal actin with phalloidin and the focal adhesion kinase (FAK) complex protein paxillin. Tendon cells isolated from Achilles tendons from healthy-aged and Sparc−/− mice were generally less spread on collagen type I and fibronectin coated surfaces (Fig. 5b/s5a), associated with the formation of large focal adhesion complexes mainly located at the cell periphery (Fig. 5a bottom). Western blot analysis of cell lysates also demonstrated an increase in the expression of paxillin in Sparc−/− and healthy-aged tendon-derived cells (Fig. 5c). Further, a profound rearrangement of the actin cytoskeleton with an increase in cortical actin was evident in healthy-aged and Sparc−/− cells (Fig. 5a top). These morphological changes are very reminiscent of what has been observed for Sparc-null mesangial cells^28^.

As it has also been demonstrated that ageing negatively affects the migration of both bone marrow stromal cells *in vivo*^29^ and tendon-derived cells *in vitro*^13^, we performed *in vitro* scratch-wound assays to determine the migratory speed of Achilles tendon-derived cells isolated from young, healthy-aged, and Sparc−/− mice. Interestingly, Sparc−/− and healthy-aged cells migrated moderately faster on collagen type I coated surfaces when compared to young cells, whereas fibronectin coating resulted in a slightly lower migration speed (Fig. S5b/c).

In summary, these results indicate that, next to moderate changes in cell migration, the decrease in Sparc expression in aged tendon-derived cells results in a more round cell morphology and the formation of more prominent paxillin containing focal adhesions. These stationary adhesions, together with an actin cytoskeleton rearrangement, potentially allow a stronger and sustained force transmission to the ECM. Indeed, seeding of *in vitro* tendon-like constructs with Sparc-null or healthy-aged tendon cells resulted in more strongly contracted constructs when compared to those seeded with young cells (Fig. 5d/e).

### Sparc regulates lipid accumulation in tendons

**Figure 6:**
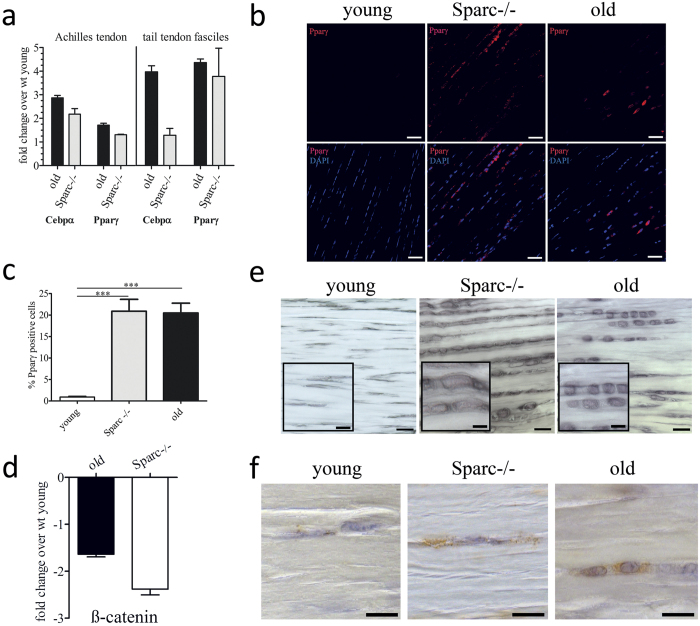
Increased lipid accretion and adipogenic marker expression in aged tendons. (**a**) mRNA levels for the adipogenic marker genes Pparγ and Cebpα were determined by quantitative RT-PCR. Shown are fold changes over mRNA levels quantified for young Achilles tendons (left) and tail tendon fascicles (right). Data represent means ± SEM of 3 independent biological replicates. (**b**,**c**) Representative immunofluorescence microscopy images and quantification of Pparγ-positive cells within the dense connective tissue of young, healthy-aged, and Sparc−/− Achilles tendons. Graphs represent mean ± SEM of percent positive cells determined for each group (n ≥ 1000 cells counted from 3 Achilles tendon samples). Scale bars: 50 μm; ***P < 0.001, One-way ANOVA (Tukey’s Multiple Comparison Test). (**d**) β-catenin gene expression in Achilles tendon tissue. Shown is fold-change in expression compared to young tendon tissue. Bars represent mean ± SEM (n = 5). (**e**) Sudan Black B staining demonstrating accumulation of lipid droplets and change in cell morphology in Sparc−/− and healthy-aged Achilles tendons. Scale bars: overview: 20 μm; insert: 10 μm; (**f**) Immunohistochemical detection of the lipid droplet membrane protein Perilipin-1 in young, Sparc−/−, and healthy-aged tendons. Scale bars: 10 μm.

Sparc has been shown to have a role in adipogenesis^30^, so we hypothesized that Sparc also influences lipid accretion in tendons. Further, several studies demonstrated that modulation of cell adhesiveness and cytoarchitecture can influence stem cell differentiation in general and adipogenesis in particular^31^,^32^,^33^. In order to characterize the potential molecular mechanisms coupling an age-dependent decrease in Sparc expression and accumulation of lipids in healthy-aged tendons, we investigated the mRNA levels of adipogenic markers in Achilles and tail tendon tissue. Indeed, in comparison to young tendon tissue the expression of both peroxisome proliferator-activated receptor γ (Pparγ) and CCAAT/enhancer-binding protein alpha (Cebpα) was increased in healthy-aged and Sparc−/− tendons (Fig. 6a). This result was further substantiated by immunofluorescence staining of mouse Achilles tendons for Pparγ, demonstrating a significant increase in cells positive for this positive regulator of adipocyte differentiation within healthy-aged and Sparc−/− tendon tissue (Fig. 6b/c). It has been previously shown that Sparc influences adipogenesis in a β-catenin dependent manner^30^ and PPARγ activity is modulated by β-catenin^34^. Consistently, we saw a reduced expression of β-catenin mRNA expression in healthy-aged and Sparc−/− Achilles tendons (Fig. 6d) and a reduced expression in healthy aged tendons was also identified by the initial SSH differential screen (Table S1). Finally, a striking increase in cells located in the dense collagenous tissue that had accumulated lipid droplets was also obvious by Sudan black B histochemical staining (Fig. 6e) and positive staining for the lipid droplet-associated protein perilipin-1 (Fig. 6f). These cells were also characterized by a spherical morphology, whereas in tendon tissue of young mice the characteristic, elongated fibroblast-like cells were observed.

These findings support the hypothesis, that the decrease in Sparc expression with age, at least in part, influences the cell phenotype in tendons, driving the differentiation of cells that have adipogenic potential and hence, an increase in lipids and lipoproteins in healthy aged tendons.

## Discussion

The musculoskeletal system undergoes significant age-related changes, contributing to physical frailty. While progressive degeneration of bone tissue and muscle loss with age is well established, the increase in occurrence of tendinopathies and tendon injuries in the elderly is less widely appreciated^35^,^36^,^37^. However, it is estimated that over one-third of persons older than 70 years suffer from some degree of tendinopathy or tendon rupture, which often is asymptomatic and increases the risk of a full tendon rupture. Tendons resemble a connective tissue rich in highly organized collagen fibers, displaying a remarkably high tensile strength. However, partly due to the low number of cells and their more or less avascular nature, tendons heal relatively slowly and their repair remains clinically challenging^38^,^39^,^40^.

Our understanding of the complex mechanisms underlying the impaired functional performance of tendon with age remains incomplete. Although tendon dysfunction has been causally linked to alterations in the mechanical properties of tendons with ageing^7^,^41^, the age-related cellular and molecular events underlying this increased risk are just beginning to unfold^13^,^22^,^42^. However, several studies based on degenerated tendon samples, reveal changes potentially due to degeneration rather than healthy ageing. Therefore, for this study, we focused on healthy mouse Achilles tendons with the aim of identifying age-related changes that may predispose tendons to degeneration. Next to the downregulation of several ECM and ECM modifying proteins, differential screening and quantitative RT-PCR revealed an age-related reduction in the expression of Sparc - Secreted protein acidic and rich in cysteine – in macroscopically normal mouse Achilles tendon tissue. Sparc, also termed osteonectin or BM-40, is a highly conserved matricellular protein that is expressed in both mineralized and non-mineralized tissues fulfilling pleiotropic effects on biological functions, including processes driving changes in cell attachment and morphology, cell motility, cell proliferation, modulation of growth factor signaling, and ECM assembly and deposition^16^. Here we show that the collagen fibril morphology of Achilles tendons in Sparc−/− mice is altered, ultimately resulting in a lower tensile strength. Overall, Achilles tendons lacking Sparc are significantly thinner compared to their age-matched wild type counterparts and seem less densely packed based on histological evaluation. Further, they are characterized by smaller fibrils, reminiscent of what has been described for the dermis of Sparc−/− mice^17^. Our findings also confirm previously published results that fibril diameters in healthy-aged wild type tendons follow a bimodal distribution with an increase in larger diameter fibrils^43^. In comparison, in Sparc−/− tendons fibril diameters remained small until 18 months of age (data not shown), indicating an impaired collagen fibril maturation in tendons in the absence of Sparc.

Similarly, collagen fibril diameter distribution did not change in aged patellar tendons for biglycan and decorin knockout mice^44^, indicating that the maturation of collagen fibrils during healthy ageing requires the concerted action of Sparc and several SLRPs in tendons.

Further, the number of fibrils was significantly increased in Sparc−/− tendons. Kalson *et al*. proposed that during embryonic tendon development the number of fibrils is determined by the number of cells residing in the tissue, which define vertical channels facilitating fibril formation, followed by an increase in fibril diameter postnatally^45^. Therefore, it seems plausible that the increase in fibril number is a consequence of an increased cellularity seen in the Sparc−/− tendon proper. Further, it was demonstrated that an increase in collagen type III results in the formation of thinner collagen I fibrils *in vitro*^46^. As in Sparc−/− tendons the expression of collagen III is dramatically increased already in young animals, a shift in the collagen I to collagen III ratio potentially also results in the formation of thinner fibrils. Interestingly, the ECM of young Sparc−/− Achilles tendons was moderately more anisotropic in comparison to their age-matched wild type counterparts. As expected, collagen fibers were less well oriented in 18 month healthy-aged tendons. Similarly, angle deviations were also greater for healthy-aged Sparc-null tendons (data not shown) based on second-harmonic generation microscopy.

Most studies indicate that an increased fibril radius is a primary determinant of improved tendon stiffness and strength^47^,^48^, which is in agreement with our experimental observations for healthy-aged wild type tendons. As expected, the ultimate tensile load is reduced in Sparc-null mice, however mechanically they showed a statistically higher elastic modulus compared to age-matched wild type flexor tendons. These results indicate that biochemical differences in the non-collagenous extracellular matrix (ECM) potentially are the causative factor for these results. However further, more elaborate biomechanical testing and tendon structure-function analyses are required to explain these findings. Moreover, it needs to be considered that aged tendons underlie long-term biochemical changes such as the deposition of advanced glycation end products (AGEs) resulting in tendon stiffening. These effects may mask other alterations caused by age-related regulation of Sparc. Overall, the ramifications of ageing-associated reduction in Sparc expression are most likely also distinct from the impact of Sparc deregulation on collagen fibril formation/maturation and on the structural and biomechanical properties of tendons. Interestingly, Sparc-null mice exhibit signs of progressive ageing, e.g. early cataract formation^49^, an osteopenic phenotype^50^, increase in adipose tissue^24^, and impaired skin integrity^17^. The limitation of adipogenesis by Sparc by inhibiting the terminal differentiation of adipocytes is of particular interest, as fatty deposits in tendons increase with age and is a sign of degeneration, potentially leading to tendon rupture^18^,^51^. Secreted Sparc regulates cell-matrix interaction and it is widely accepted that the modulation of cell-surface receptors and the ECM gives rise to complex physiological phenomena, including cell differentiation. Furthermore, hallmarks of adipocyte differentiation include changes in cell shape and ECM remodeling^52^. Indeed, not only did we observe a change in cell shape *in vitro*, but the expression of peroxisome proliferator-activated receptor γ (PPARγ) in 18 month old tendons and Sparc-null tendons also significantly increased *in vivo*. Most importantly, we were able to demonstrate the increased presence of perilipin-1 in healthy-aged and Sparc−/− tendon proper, demonstrating the accumulation of lipid droplets. Therefore, our data suggest that the age-related decrease in Sparc modulates the cell-ECM interaction of tendon-resident stem and progenitor cells and together with a change in ECM properties favors adipocyte differentiation.

In conclusion, our findings provide new mechanistic insights into the molecular and cellular events associated with tendon ageing that may help pin down the underlying causes behind the increasing incidence of tendon dysfunction in the elderly.

## Materials and Methods

### Animals and cell culture

Three month (young) and 18 month old (healthy-aged) male C57BL/6JRj (Janvier Labs rodent research models and associated services, Saint Berthevin, France) mice were used for all experiments. The generation of Sparc−/− mice has been previously described^53^ and breeding pairs were kindly provided by Prof. Stephane Heymans (Maastricht University, Maastricht, The Netherlands). All animal experiments and procedures were conducted in accordance with Austrian laws on animal experimentation and were approved by Austrian regulatory authorities (Permit No. BMWFW-66.012/0013-WF/V/3b/2016).

Achilles tendons were dissected under sterile conditions and washed in sterile PBS without Ca^2+^ and Mg^2+^ (subsequently referred to as PBS) and α-MEM (minimal essential medium with 2 mM GlutaMAX™). Tendon tissue was cut into small pieces and incubated in 3 mg/ml Collagenase Type II (Gibco Lifetechnologies, Vienna, Austria) in α-MEM with 10% fetal bovine serum (FBS), 2 mM GlutaMAX™ (both ThermoFischer Scientific, Vienna, Austria), 100 units penicillin and 0.1 mg ml^−1^ streptomycin (P/S, ThermoFischer Scientific, Vienna, Austria), O/N at 37 °C, 5% CO_2_ and 90% humidity. The following day, tendon stem and progenitor cells (subsequently referred to as TDSPCs) were washed in α-MEM with 10% FBS and P/S and cells were cultivated until near confluency. Cell number and cell viability were determined with a Luna™-fl cell counting system (Logos Biosystems, Annandale, USA) according to the manufacturer’s instructions. For all *in vitro* experiments TDSPCs were isolated and pooled from at least 5 animals for each group and experiments were performed with cells at passage 3 and at least in triplicates.

### RNA isolation, quantification and validation

Mouse Achilles and tail tendons were homogenized in TRI Reagent^®^ (Sigma-Aldrich, Vienna, Austria) on ice using an Ultra-Turrax (IKA, Staufen, Germany). Total RNA was prepared according to manufacturer’s recommendations with minor modifications. Two additional chloroform extraction steps were performed, and before the last chloroform extraction step a DNase I digestion (MBI Fermentas, ThermoFischer Scientific, Vienna, Austria) was performed. Subsequently, total RNA was precipitated for 30 min at −20 °C with an equal volume of ice cold isopropanol followed by centrifugation for 30 min at 13.000 rpm at 4 °C. RNA pellets were washed in 75% EtOH, air dried and resuspended in RNase-free water supplemented with 20 units Ribolock RNase inhibitor (ThermoFischer Scientific, Vienna, Austria) and stored at −80 °C until further use.

TDSPCs were washed in PBS and lysed in ice cold TRI Reagent^®^. Total RNA was prepared as described above.

RNA yield was quantified using a Nanodrop 2000C (ThermoFisher Scientific, Vienna, Austria) and RNA integrity was verified using an Experion Automated Electrophoresis system (Biorad, Munich, Germany). A minimum requirement of the RNA quality indicator (RQI) >7.5 was chosen.

### Subtractive suppression hybridization

Total RNA of 10 animals each (3 months and 18 months old male C57BL/6 mice) was pooled to a total amount of 1 μg (100 ng each) and transcribed with the SMARTer cDNA synthesis kit (Takara Bio Europe/SAS, Saint-Germain-en-Laye, France) according to the manufacturer’s instructions. Differentially expressed cDNAs were identified and isolated using the PCR-Select cDNA Subtraction Kit (Takara Bio Europe/SAS, Saint-Germain-en-Laye, France) and PCR Differential Screening Kit (Takara Bio Europe/SAS, Saint-Germain-en-Laye, France). Subtracted cDNA libraries were cloned into the pCR2.1-TOPO vector and transformed into TOP 10F′ chemically competent E. coli cells (ThermoFisher Scientific, Vienna, Austria). Bacterial clones were grown O/N at 37 °C on LB-agar plates supplemented with 50 μg/ml Kanamycin, 50 μg/ml Ampicillin, 100 μM IPTG and 40 μg/ml X-gaL. All white colonies were isolated and transferred to LB-agar plates with respective antibiotics.

Digoxigenin-labeled probes were synthesized and purified as described in the manual for the PCR DIG-labelling mix (Roche Diagnostics, Vienna, Austria). For hybridization, replicas of colonies were transferred to 4 nylon membranes (Biorad, Munich, Germany; 1 membrane each for hybridization with the (i) forward subtracted probe, (ii) unsubtracted tester probe, (iii) reverse subtracted probe, and (iv) unsubtracted driver probe, followed by denaturation in 0.5 M NaOH, 1.5 M NaCl, neutralization in 0.5 M Tris-HCl pH 7.5, 1.5 M NaCl and cross-linking by incubation at 80 °C for 1½ hours. For pre-hybridization, membranes were incubated in 5× SSC (standard saline citrate buffer; 750 mM NaCl, 75 mM tri-sodium citrate dihydrate pH 7.0), 0.02% SDS, 1% blocking reagent (Roche Diagnostics, Vienna, Austria) for 4 h at 72 °C with continuous agitation. Prior to hybridization, digoxigenin-labeled probes were denatured at 95 °C for 5 min, and 25 μl probe was added to the pre-hybridization solution. Hybridization was carried out O/N at 72 °C with continuous agitation. Membranes were then washed twice for 15 min in 2× SSC + 0.1% SDS at room temperature, twice in 2× SSC + 0.1% SDS at 68 °C (low stringency) and twice in 0.2× SSC + 0.1% SDS at 68 °C (high stringency). For detection of digoxigenin-labeling, membranes were incubated for 5 min at RT in Maleate buffer (100 mM maleic acid pH 7.5, 150 mM NaCl), followed by blocking membranes for 1 h at room temperature in Maleate buffer with 0.5% Tween-20 and 1% blocking reagent and incubation with anti-digoxigenin-POD Fab fragments (Roche Diagnostics, Vienna, Austria; 1:1000) in Maleate buffer with 0.5% Tween-20 and 1% blocking reagent for 1 h at RT. Membranes were then rinsed twice and washed 3 times for 5 min in Maleate buffer with 0.5% Tween-20. Finally, membranes were equilibrated in TBS (Tris buffered saline, 100 mM Tris-HCl pH 7.5, 150 mM NaCl) and incubated with Clarity Western substrate (Biorad, Munich, Germany) as described in the manufacturer’s protocol. Chemiluminescence was detected with the ChemiDoc MP imaging system (Biorad, Munich, Germany). Identification of differentially expressed clones was performed according to the instructions in the PCR-Select Differential Screening Kit user manual (Takara Bio Europe/SAS). Respective clones were isolated and sequenced, and functional annotation was performed with the Database for Annotation, Visualization and Integrated Discovery (DAVID)^19^.

### Reverse transcription and quantitative PCR

The same total RNA pools used for subtractive hybridization were reverse transcribed with iScript RT-PCR Supermix (Biorad, Munich, Germany). 5 ng/well cDNA were subsequently analyzed by quantitative PCR using TaqMan gene expression mastermix (Applied Biosystems, ThermoFischer Scientific, Vienna, Austria), and TaqMan assays purchased either from IDT (Coralville, IA, USA) or Applied Biosystems (ThermoFisher Scientific, Vienna, Austria) (see Table S2). Amplification conditions were 50 °C for 2 min, 95 °C for 10 min, followed by 40 cycles of 95 °C for 15 s and 60 °C for 1 min. All samples were run in duplicate. CQ values were analyzed using qBasePlus v. 2.4 (Biogazelle NV, Zwijnaarde, Belgium) and normalized relative quantities were calculated by normalizing the data to the expression of previously validated endogenous control genes as described by Vandesompele *et al*. (2002). The normalized quantities were then determined for the candidate genes scaled against the expression values determined for the young animals to generate fold changes in expression.

### Protein lysates, SDS-PAGE and Western Blot, collagen extraction and quantification

TDSPCs, isolated of at least 5 animals per preparation, were washed with PBS and lysed in ice cold lysis buffer (20 mM Tris-HCl pH 7.4, 0.1% SDS, 150 mM NaCl, 10 mM EDTA, 0.1% Nonidet P-40, protease inhibitors AEBSF at 1.04 mM, Aprotinin at 0.8 μM, Bestatin at 40 μM, Leupeptin at 20 μM, E-64 at 14 μM, and Pepstatin A at 15 μM). Five individual protein lysate preparations were made.

Fascicles dissected from tail tendons of 5 indiviual animals for each group were washed twice in ice cold PBS and flash frozen in liquid nitrogen. Following homogenization with a mortar and pestle, ground tissue was resuspended in lysis buffer, incubated on a shaker for 15 min on ice and centrifuged at 12.000 g for 15 min at 4 °C to remove cell debris. Three individual protein lysates were prepared. Protein content was determined with the BCA protein quantification kit (Pierce, ThermoFisher Scientific, Vienna, Austria).

Ten to 20 μg of total protein of the TDSPC or tendon fascicle lysate were separated on 10–12% SDS-polyacrylamide gels in Laemmli buffer^54^. Proteins were then transferred to a PVDF membrane (Biorad, Munich, Germany) using 3 mM Na_2_CO_3_, 10 mM NaHCO_3_, 20% methanol and 0.0375% SDS for 2 h at 40 V and 4 °C. Membranes were blocked in 5% non-fat dry milk powder, 5% BSA, or 5% casein hydrolysate in TBS with 0.5% Tween-20, respectively for 2 h at RT. Membranes were incubated O/N at 4 °C in blocking solution containing primary antibodies (see Table S2 for dilutions), followed by washing in TBS containing 0.5% Tween-20 (3 × 5 min and 2 × 15 min) and incubation with horseradish peroxidase (HRP) conjugated secondary antibodies (see Table S2) diluted in TBS containing 0.5% Tween-20 for 1 h at RT. Detection was performed with Clarity Western ECL Substrate and the Chemidoc MP imaging system (both Biorad, Munich, Germany). Relative densitometric intensities of at a minimum of 3 individual experiments were determined using ImageLab 5.2 software (Biorad, Munich, Germany).

For total collagen extraction 10 mg of dry weight tendon powder was resuspended and hydrolyzed in 100 μl 6N HCl for 18 h at 120 °C. Then collagen solution was cooled to room temperature and neutralized with 1 volume 6N NaOH. Collagen content was determined by photometric quantification of hydroxyproline. In brief, 350 μl chloramine-T solution (23.5 mg chloramine-T per ml water) were mixed with 50 μl collagen sample and incubated for 25 min at RT. Then 500 μl Ehrlich’s solution (750 mg 4-(Dimethylamino)-benzaldehyd, 3 ml 1-propanol, 1.3 ml 70% perchloric acid filled up to 10 ml with water) were added, followed by an incubation at 65 °C for 20 min. Absorbance was measured at 560 nm and hydroxyproline concentration was determined using a hydroxyproline standard curve.

### Biomechanical testing

For biomechanical testing, digital flexor tendons were isolated from 3 month old male wild type mice (n = 23), and from 3 month old male SPARC−/− C57BL/6 mice (n = 16) and from 18 month old male wild type C57BL/6 mice (n = 17). Immediately after isolation, tendons were wrapped in gauze soaked with PBS to prevent drying and stored at −20 °C until testing. Before measurement, tendons were clamped in a custom made fixation device and tendon diameter was measured using a digital caliper. Specimens were tested on a universal material testing machine (Zwick/Roell 500N Zwicki, Ulm/Einsingen, Germany). After application of a preload of 0.1N samples were loaded at a speed of 0.1 mm/min until failure. Stiffness was calculated from the linear proportion of the force/elongation curve and is expressed as N/mm. Tendon material properties were calculated by converting the load-displacement data into stress-strain data using the measured tendon diameter and elongation during testing. Ultimate stress was defined as the stress at tendon failure. Elastic modulus was calculated as the slope of the linear region of the stress-strain curve. Samples, which had partially slipped during tension testing were excluded from the analysis.

For statistical analysis, maximum tensile load, stiffness, ultimate stress and elastic modulus were analyzed by One way ANOVA and Tukey’s Multiple Comparison Test.

### Histology, immunohistochemistry and immunofluorescence

Mouse hind limbs were fixed in 4% paraformaldehyde for at least 18 h at 4 °C, washed in PBS, dehydrated in a graded ethanol series and finally paraffin embedded. Tissue sections (5–7 μm) for histological and immunohistological stainings were deparaffinized in Rotihistol (Carl Roth, Karlsruhe, Germany), rehydrated in a graded ethanol series and equilibrated in PBS. Prior to the detection of Perilipin-1, tissue sections were incubated in tri-sodium citrate-buffer (containing 2.94 g per liter A. dest. pH 6.0) for 3 h at 60 °C, cooled to RT and equilibrated in PBS and processed for immunodetection. For immunohistological detection of Sparc tissue sections were incubated in 0.5% saponin for 30 min at RT, blocked with Roti-Immunoblock (Carl Roth, Graz, Austria; for Sparc) or horse serum (VectorLabs, Szabo-Scandic, Vienna, Austria; for Perilipin 1) for 30 min at RT followed by primary antibody incubation (antibodies and dilutions, see Table S2) O/N at 4 °C. Subsequently, tissue sections were washed in PBS with 0.5% Tween-20 and endogenous peroxidase was quenched with 3% H_2_O_2_ in methanol for 30 min at RT. Incubation of secondary peroxidase-conjugated antibodies (see Table S2) was performed for 30 min at RT. Finally, slides were washed in PBS with 0.5% Tween-20 and peroxidase activity was detected with 3,3–diaminobenzidine tetrahydrochloride (DAB, Sigma-Aldrich, Vienna, Austria) and stopped in PBS with 0.5% Tween-20. Tissue sections were counterstained with Novocastra Hematoxylin (Leica, Vienna, Austria) for 2 min and mounted with Roti-Histokit (Carl Roth, Karlsruhe, Germany). Sections from 14 healthy-aged and 12 young Achilles tendons were evaluated and a minimum of 2500 cells in total were counted. Quantitative cell counts were performed using ImageJ (v. 1.50a).

Collagen fibers were visualized by Herovici’s polychrome staining. Briefly, after deparaffinizing, tissue sections were rehydrated in a graded ethanol series, equilibrated in water and nuclei were stained with Novocastra Hematoxylin (Leica, Vienna, Austria). Then sections were incubated in polychrome solution (2 parts 0.1% van Gieson solution plus 1 part 0.05% methylblue in 1% acetic acid) for 5 min at RT, followed by incubation for 1 min in 1% acetic acid, 4 × 1 min in 100% ethanol, and 2 × 2 min in Rotihistol and mounted with Roti-Histokit (both Carl Roth, Karlsruhe, Germany). For Sudan Black B staining tissue sections were deparaffinized and rehydrated to 70% ethanol. Then sections were incubated in Sudan Black B solution (0.7 g Sudan Black B in 100 ml 70% ethanol) for 8 min at RT, followed by rinsing and differentiation in 50% alcohol until background was pale gray. Finally, tissue sections were washed in water, counterstained with Nuclear fast red and mounted in Roti-mount aqua (both Carl-Roth, Karlsruhe, Germany).

For immunofluorescence staining cells were fixed with 4% paraformaldehyde for 20 min at room temperature. Cells were washed three times with PBS and permeabilized with 0.1% Triton X-100 and 1% bovine serum albumin in PBS for 1 h at RT. Phalloidin-ATTO565 or primary antibodies against Tenomodulin, SPARC, paxillin, and Pparγ were diluted in the same buffer (antibodies and dilutions see Table S2) and samples were incubated O/N at 4 °C. After 3 washing steps with PBS, the cells were incubated with fluorescently labelled secondary antibodies (see Table S2) and DAPI for 1 h at RT. After a final 3 washing steps with PBS, the cells were mounted with Prolong Gold antifade mounting medium (ThermoFischer Scientific, Vienna, Austria). The stained cells were analyzed with a Zeiss LSM710 laser scanning microscope (Carl Zeiss GmbH, Munich, Germany) and for quantitative analysis tissue sections of 10 individual mice were used and a minimum of 1000 cells were counted using ImageJ (v. 1.50a).

### Transmission electron microscopy and fibril diameter measurements

For ultrastructural analysis, Achilles tendons from healthy-aged and young wild type and Sparc−/− mice, (5 animals per group), were dissected out and processed for transmission electron microscopy as previously described^55^ with minor modifications. In brief, samples were incubated in primary fixative (100 mM sodium phosphate buffer pH 7.0 containing 2% glutaraldehyde for 30 min at RT, then transferred to fresh primary fixative and incubated for another 2 h at 4 °C. After several washes in 200 mM sodium phosphate buffer [pH 7.0], specimens were post-fixed in secondary fixative (50 mM phosphate buffer pH 6.2 containing 1% glutaraldehyde and 1% osmium tetroxide; Electron Microscopy Sciences, Hatfield, USA) for 40 min at 4 °C. Subsequently, specimens were thoroughly washed with distilled water and en bloc stained in 1% aqueous uranyl acetate for 16 h at 4 °C. Samples were dehydrated in graded ethanol series (30%, 50%, 70%, 80%, 95%) followed by four changes of 100% ethanol for 15 min each at RT. Samples were then gradually infiltrated with a mixture of low viscosity resin (UltraBed Kit, Electron Microscopy Sciences) and ethanol according to the manufacturer’s instructions. Finally, samples were placed in embedding moulds filled with pure resin and polymerized at 60 °C for 24 h. Ultrathin sections (80 nm) were cut on a Reichert-Jung Ultracut ultramicrotome(Leica Microsystems, Vienna, Austria). Sections were mounted on Formvar coated 75-mesh copper grids, contrasted with aqueous solutions of uranyl acetate and lead citrate, post-stained with 0.5% uranyl acetate and 3% lead citrate and analyzed with a Zeiss EM 910 electron microscope equipped with a Troendle sharp:eye 2 k CCD camera (Carl Zeiss GmbH, Oberkochen, Germany.

### Second-harmonic generation (SHG)-microscopy and data analysis

Mouse hind limbs were fixed as described above. Tissue sections (30 μm) were deparaffinized in Rotihistol (Carl Roth, Karlsruhe, Germany), rehydrated in a graded ethanol series and equilibrated in PBS and mounted in Fluoromount-G^®^ (Southern Biotech, Birmingham, AL, USA). SHG microscopy was performed using a Zeiss 780 NLO multiphoton microscope (Carl Zeiss Microscopy GmbH, Munich, Germany).

Morphological collagen data were extracted from 3D images (z-stack) by combining Fourier power spectrum analysis and wedge filtering, as previously described^27^,^56^. The analysis resulted into discrete angular distributions of relative amplitudes which resembled the fiber orientations. Collagen fiber orientations were then fitted using a π-periodic von Mises distribution ρ which was assumed to be symmetrical around Φ = 0, i.e.


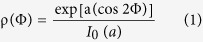


with the so-called concentration parameter a, determining the shape of the von Mises distribution and I0(a) denoting the modified Bessel function of the first kind of order 0, given by


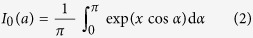


For a = 0 we have ρ(Φ) ≡ 1, which corresponds to an isotropic fiber distribution, whereas for a → ∞, ρ(Φ) becomes a delta function which corresponds to perfect fiber alignment.

### Wounding assays and cell attachment/area

Six well plates or cover slips were coated with collagen type I from rat tails (Sigma-Aldrich, Vienna, Austria) with a final concentration of 10 μg/cm^2^ for 2 h at 37 °C. For fibronectin coating (Sigma-Aldrich, Vienna, Austria), dishes and coverslips were incubated for 1 h at 37 °C at a final concentration of 20 μg/ml. Subsequently, excess solution was removed, plates and cover slips were air dried, and prior to seeding plates were washed with sterile water and sterile PBS.

For cell area measurements TDSCPs were stained with phalloidin-ATTO565 (see above for details) and cell area was determined for a minimum of 160 cells using ImageJ (v. 150a).

For wounding assays, culture-inserts (ibidi, Planegg/Martinsried, Germany) were mounted to uncoated, collagen type I or fibronectin 1 coated 6-well plates. TDSPCs (passage 3) were resuspended in α-MEM with 10% FBS, 2 mM GlutaMAX and P/S to a final cell number of 4.5E + 5 cells/ml. Seventy μl of this TDSPCs suspension were seeded per Culture-Insert well and let adhere for at least 6 h at 37 °C, 5% CO_2_ and 90% humidity. Then medium was changed to α-MEM with 2% FBS, 2 mM GlutaMAX and P/S and cells incubated O/N at 37 °C, 5% CO_2_ and 90% humidity. One hour before time lapse microscopy culture-inserts were removed, medium was again changed to α-MEM with 10% FBS, 2 mM GlutaMAX^®^ and P/S and cells were allowed to migrate towards each other. Time lapse was performed 12 frames per hour for 24 h on a Nikon Eclipse Ti Invert microscope equipped with an Okolab Bold Line stage top chamber. Time lapse videos were acquired with a CCD camera (ANDOR-Clara DR-328G-CO2-SIL; Sony) using NIS elements (vers. 4.30.02, Nikon GmbH, Vienna, Austria) and were subsequently analyzed using ImageJ (v. 150a). Five biological replicates were analysed.

### *In vitro* tendon construct formation

*In vitro* tendon constructs from mouse TDSPCs were assembled as described in refs 57 with some modifications. Each well of a 6-well cell culture plate was coated with 1 ml Sylgard silicone (Dow-Chemicals, Vienna, Austria) and allowed to cure at 48 °C O/N. Subsequently, two 1 cm silk sutures were pinned with minutien insect pins (0.1 mm diameter, Science Services, Munich, Germany) onto the silicone layer with 1 cm distance between the two sutures. Six-well plates were sterilized by incubating in 70% ethanol and exposing to UV-irradiation for 30 min each. Before use, the plates were washed with sterile PBS. TDSPCs (passage 3) were resuspended in 4 mg/ml fibrinogen and 1 unit thrombin in serum-free α-MEM with 2 mM GlutaMAX™ to a final cell number of 2.5E + 5/ml. Cell suspension was immediately spread over the complete silicone coated surface and fibrin gel was allowed to set for 1 h at 37 °C. The gel was then carefully loosened from the edges and overlaid with α-MEM with 10% FBS, 2 mM GlutaMAX™, P/S, 0.2 mM ascorbic acid, 0.05 mM L-proline and 10 μg/ml Aprotinin and cultured until the fibrin matrix was fully contracted between the 2 suture ends. Every other day the medium was changed and the fibrin gel detached with a small pipet tip if necessary. All tendon constructs were formed within 21 days of contraction and were kept in culture for additional 21 days; finally the diameters of the formed constructs were measured from digital images using ImageJ (v. 1.50a). The diameters of a total of 15 constructs per TDSCP group (SPARC−/−, old, young) were determined.

## Additional Information

**How to cite this article**: Gehwolf, R. *et al*. Pleiotropic roles of the matricellular protein Sparc in tendon maturation and ageing. *Sci. Rep.* **6**, 32635; doi: 10.1038/srep32635 (2016).

## Supplementary Material

Supplementary Information
