# Supplementary material for: Pleiotropic roles of the matricellular protein Sparc in tendon maturation and ageing
Source: Sci Rep. 2016 Sep 2;6:32635. doi: 10.1038/srep32635 (PMC5009305; doi:10.1038/srep32635)
Supplement: Supplementary Information [file srep32635-s1.pdf]

Pleiotropic roles of the matricellular protein Sparc in tendon maturation and ageing.

Renate Gehwolf [1,2], Andrea Wagner [1,2], Christine Lehner [1,2], Amy D. Bradshaw [3], Cornelia Scharler [4], Justyna A. Niestrawska [5], Gerhard A. Holzapfel [5], Hans-Christian Bauer [1,2], Herbert Tempfer [1,2], Andreas Traweger* [1,2]

FIGURE LEGENDS: Supplementary figures

Figure S1. Differential gene expression in mouse Achilles tendon tissue

(a) Quantitative RT-PCR results demonstrating repression of Matrix metalloproteinases 2 and 9 and the tendon cell associated marker Mohawk in healthy-aged mouse Achilles tendons. A change in expression ≥2.0 was assumed as a significant change (n=5 Achilles tendon samples); graphs represent means±SEM. (**b**) Repression of the above-mentioned genes is also seen in in tendon progenitor/stem cells of healthy-aged mouse Achilles tendons *in vitro* (passage nr. 3). qPCR data for healthy-aged tendons were normalized to young tendons, a fold change ≥2.0 was assumed as a significant difference in expression; shown are means±SEM.

Figure S2. Collagen type I to type II mRNA ratio

Comparison of collagen type I to type III mRNA ratios for young, Sparc-/-, and healthy-aged Achilles tendons. Data represent means±SEM of 3 independent biological replicates; ***P < 0.001, One-way ANOVA (Tukey's Multiple Comparison Test).

Figure S5. Cell area and cell migration

(a) Cell area of Sparc-/- TDSPCs on fibronectin coated surfaces is significantly reduced compared to young and healthy-aged wild type cells. No significant differences between young and healthy-aged tendon cells are seen. Cell area of a minimum of 160 cells was measured, ***P < 0.001, **P<0.01, n.s. = not significant, (Kruskall-Wallis test with Dunn's Multiple Comparison test). (**b, c**) Wound healing assays on collagen type I and fibronectin coated surfaces reveal moderate changes in cell migration

speed *in vitro* for Sparc-/- and healthy-aged cells migrate moderately faster *in vitro*. Dot blots show results from 3 independent experiments and red bar indicates mean. Representative images for scratch-wound assays 0h and 10h after creating the wound are shown. ***P < 0.001, **P < 0.01, One-way ANOVA (Tukey's Multiple Comparison Test).

Supplementary Figures

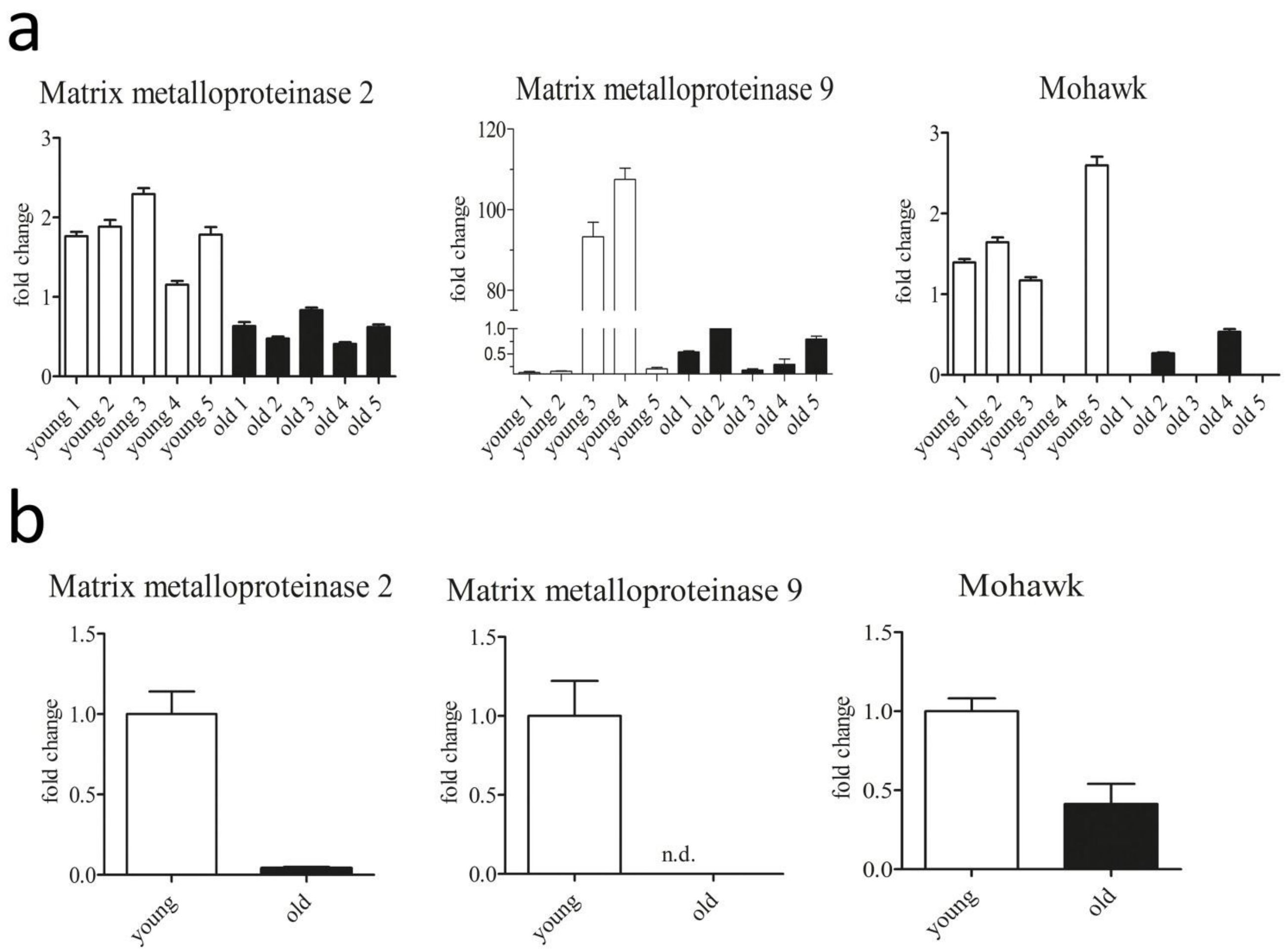

Suppl Figure 1

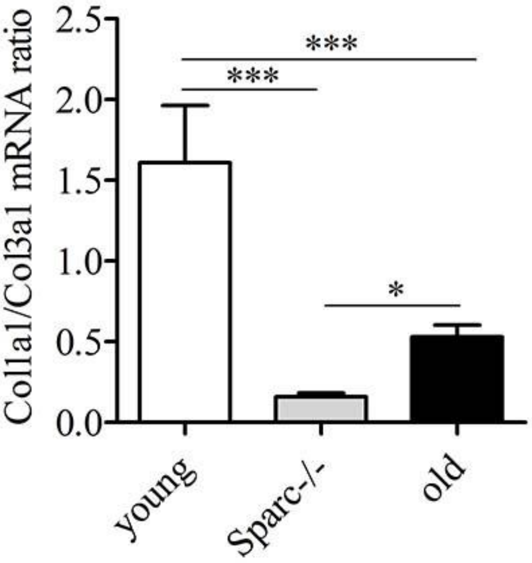

Suppl Figure 2

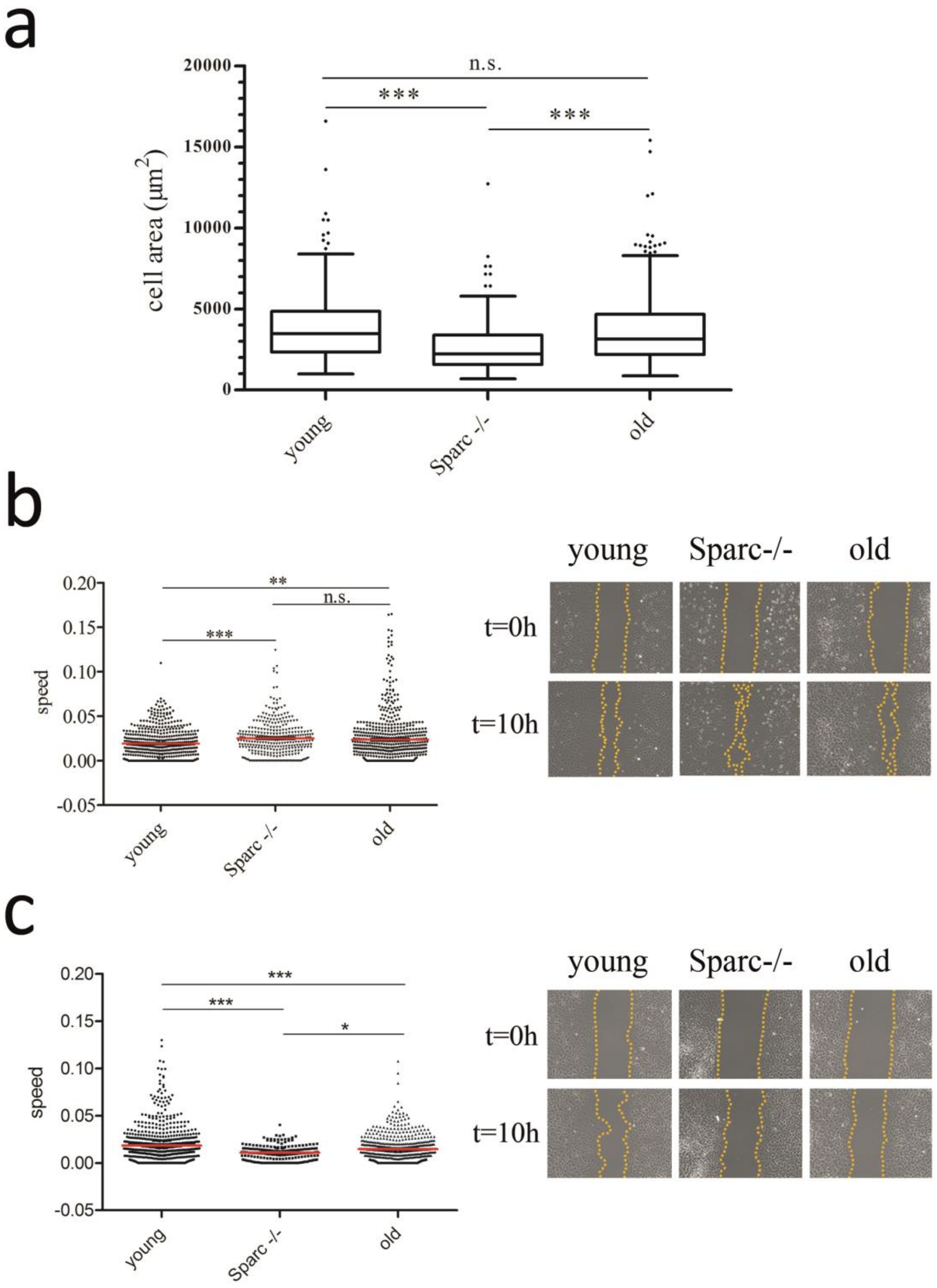

Suppl Figure 5
